# Associations between access to alcohol outlets and alcohol intake and depressive symptoms in women from socioeconomically disadvantaged neighbourhoods in Australia

**DOI:** 10.1186/s12889-017-4022-4

**Published:** 2017-01-17

**Authors:** Karen E. Lamb, Lukar E. Thornton, Megan Teychenne, Catherine Milte, Ester Cerin, Kylie Ball

**Affiliations:** 1 Deakin University, Institute for Physical Activity and Nutrition, School of Exercise and Nutrition Sciences, Geelong, VIC Australia; 2 Institute for Health and Ageing, Australian Catholic University, Melbourne, Australia; 3 The University of Hong Kong, School of Public Health, Pokfulam Road, Hong Kong, China

**Keywords:** Alcohol, Alcohol outlet access, Harmful consumption, Depressive symptoms, Area-level disadvantage

## Abstract

**Background:**

This study examined associations between alcohol outlet access and alcohol intake, depressive symptoms score and risk of depression among women residing in disadvantaged neighbourhoods in Victoria, Australia.

**Methods:**

Data on depressive symptoms, alcohol intake and socio-demographic characteristics were obtained from a sample of 995 adult women from Victoria, Australia who were surveyed as part of the Resilience in Eating and Activity Despite Inequality (READI) study. The location of all licensed alcohol outlets in Victoria was obtained from the Victorian Commission for Gambling and Liquor Regulation. Participant and alcohol outlet addresses were geocoded to calculate individual alcohol outlet access, defined as the number of outlets (all and by sub-type) within 0.4 km and 3 km of participants’ homes. Separate regression models with clustered standard errors were fitted to examine associations between access and alcohol intake according to national recommended limits for short- and long-term harm, frequency of consumption above long-term harm guidelines, depressive symptoms score and risk of depression.

**Results:**

Odds of consumption within short-term harm guidelines (≤4 drinks on any day) decreased with increasing access within 3 km, irrespective of outlet type. Typically, there was no evidence to support associations between access and consumption above long-term harm guidelines (>2 drinks on any day) unless considering frequency of consumption at this level where results showed decreased odds of ‘don’t drink’ versus frequently drinking above long-term harm guidelines (i.e., >2 drinks at least once per week) with increasing access at either distance. Although there was no evidence of an association between any of the alcohol outlet access measures and depressive symptoms score, odds of being at risk of depression decreased with increasing access within 3 km.

**Conclusions:**

This study found some evidence to support an association between increasing alcohol outlet densities of all types and harmful levels of alcohol consumption, and the association appears to be dependent on the distance threshold considered, among women residing in socioeconomically disadvantaged neighbourhoods within Victoria, Australia. However, higher numbers of alcohol outlets appear to be associated with a slightly lower risk of depression, with further research needed to identify the direction and mechanisms underlying this unintuitive association.

## Background

High levels of alcohol consumption are associated with a number of adverse health outcomes including cardiovascular disease, liver cirrhosis, certain cancers, and mental health issues such as depression [1]. Almost one fifth of Australians consume more than two standard drinks on any day, exceeding lifetime risk guidelines [2], while a tenth of Australian women report exceeding these guidelines [3]. Alcohol consumption and depression are linked, with increased alcohol consumption associated with higher levels of depression, although this relationship is complex and bi-directional [4]. Depression is an increasingly prevalent mental illness [5] which can severely impact physical and psychosocial health [6, 7]. Projections suggest this is likely to be the second leading cause of disease burden worldwide by 2030 [8]. Importantly, research has shown that women and those experiencing socioeconomic disadvantage (at the individual- or area-level) are at an increased risk of depression [9–11]. In Australia, the prevalence of depression in the population is estimated to be around 10%, with more women than men affected [12].

There has been growing interest in the effect of alcohol access on alcohol consumption with evidence mostly suggestive of a positive association [13, 14]. However, a recent review highlighted inconsistencies in the evidence linking alcohol outlet access around the home to alcohol use [15]. That review recommended that future studies consider fine-grained measures of alcohol outlet access rather than simply total numbers of outlets. For example, it was suggested to test sub-categories of alcohol outlets separately (e.g., premises which allow alcohol consumption on-site; outlets which sell packaged alcohol to be consumed elsewhere) and to also test potential non-linear associations between outlet access and outcomes [15].

Prior work demonstrates that various aspects of the neighbourhood built environment, such as access to healthy food stores, health services, and parks, are associated with mental health outcomes such as depressive symptoms, with results suggesting a protective effect of access to these services [16, 17]. Although there is potential for access to alcohol to also impact health conditions such as depressive symptoms, this has been less studied. Greater alcohol availability could plausibly be linked with poorer mental health of residents via several pathways, including increasing the likelihood of excessive consumption or through social problems associated with living in an area characterised by high alcohol consumption of others. To our knowledge, only one study in Australia has explicitly considered associations between alcohol outlet access and depression, finding some evidence of an association, although this attenuated after confounder adjustment [18].

To better understand the role of alcohol outlet access on both alcohol consumption and mental health, this study utilised fine-grained alcohol outlet access measures to better understand associations between access and these two outcomes. By considering women living in disadvantaged neighbourhoods, this study targets a group at high risk of poor mental health.

## Methods

Individual data were obtained from the Resilience in Eating and Activity Despite Inequality (READI) cohort study which sampled women residing in disadvantaged urban and rural suburbs of Victoria, Australia in 2007/08. Full details of the recruitment and retention to Wave II are provided elsewhere [19], with sample sizes noted below. The same follow-up procedure was used in Wave III. In brief, suburbs were defined as urban or rural using a classification of cities, fringe and rural areas consistent with the Australian Regional Infrastructure Development Fund Act. Within the urban (metropolitan Melbourne, rural cities, all areas within 10 km radius of these cities, areas within a 10 km radius of other Victorian cities with a population of 20,000) and rural (outside metropolitan Melbourne and outside 25 km radius of rural cities) strata, suburbs were classified according to the 2001 Australian Bureau of Statistics Index of Relative Socioeconomic Disadvantage (IRSD). Forty suburbs from the bottom third of the IRSD classification were randomly sampled from within the urban and rural strata. One hundred and fifty women aged between 18 and 45 years within each of the 80 suburbs were randomly identified from the Australian electoral roll (it is compulsory for Australian citizens to register in the electoral roll).

Participants responded to mail-out questionnaires including measures of health behaviours, including alcohol intake and depressive symptoms, and sociodemographic characteristics at three waves. At Wave I, 4349 women (39% of those delivered a survey) completed the questionnaire. Participants who consented to follow-up and who remained eligible (i.e., continued to reside in a READI suburb) were contacted in 2010/11 to complete Wave II surveys with surveys completed by 1912 eligible participants. A third wave of survey data was collected in 2013/14. Of 4349 women who participated in Wave I, 1560 (36%) remained at Wave III. This study only used Wave III data to ensure a temporal match between the survey data and alcohol outlet data. Three women declined to participate further after the Wave III survey and were not contacted about this study.

Of 1557 women contacted, 1240 (80%) were included after omitting those who declined to have their address mapped (*n* = 7), who no longer lived in a READI suburb (*n* = 129), or did not supply a valid street address (*n* = 181). Given recommendations to avoid consuming alcohol during pregnancy, only women who were not pregnant at Wave III (*n* = 1188) were considered. In addition, individuals who had missing outcome data were excluded (alcohol consumption: *n* = 4; depressive symptoms: *n* = 9). The analysis was restricted to participants who had remained at the same address at all waves (*n* = 995). Sensitivity analyses examined participants who had moved house between Wave I and Wave III but remained in READI suburbs (*n* = 1175). Deakin University Human Research Ethics Committee granted ethics approval.

### Outcome measures

#### i) Alcohol intake

Participants were asked: “Over the last 12 months, on days when you were drinking alcohol, about how many glasses of beer, wine and/or spirits altogether did you usually drink?” (categories: don’t drink alcohol/1/2/3/4/5/6/7/8/9/10+ glasses per day) and “Over the last 12 months, on average how often did you drink beer, wine and/or spirits?” (categories: <once per month/1–3 days per month/1 day per week/2 days per week/3 days per week/4 days per week/5 days per week/6 days per week/every day). According to Australian National Health and Medical Research Council guidelines for reducing health risks, women should consume no more than four standard alcoholic drinks on a single occasion to reduce alcohol-related injury risk and no more than two alcoholic drinks on any day to reduce risk of harm from future alcohol-related disease [20]. Therefore, two outcomes linked to these guidelines were considered: 1) ‘short-term harm’ categorised as: don’t drink; ≤4 glasses per drinking occasion; >4 glasses per drinking occasion and 2) ‘long-term harm’ categorised as don’t drink; ≤2 glasses per drinking occasion; >2 glasses per drinking occasion. To incorporate frequency, we considered a third outcome which combined the two alcohol consumption questions: don’t drink; drink quantities within long-term harm guidelines less than once per week (termed ‘infrequent drinker within guidelines’); drink quantities above long-term harm guidelines less than once per week (termed ‘infrequent drinker above guidelines’); drink quantities within long-term harm guidelines at least once per week (termed ‘frequent drinker within guidelines’); drink quantities above long-term harm guidelines at least once per week (termed ‘frequent drinker above guidelines’).

#### ii) Depressive symptoms

Depressive symptoms were assessed using the 10-item version of the Center for Epidemiologic Studies Depression Scale (CESD-10), a widely used and validated measure of depression risk [21, 22]. The CESD-10 includes statements relating to depressive symptoms experienced in the past week (e.g., “I was bothered by things that don’t usually bother me”; “I felt that everything I did was an effort”) and participants rated themselves using a 4-point ordinal scale ranging from rarely or none of the time (<1 day), to most/all of the time (5–7 days). Ordinal responses were given a score (from 0 to 3) and scores for the 10 items were summed and analysed as both a continuous variable, with higher scores indicating increased risk of depression, and a binary variable where a CESD-10 score ≥10 indicated being classified as ‘at risk’ of depression [22].

### Exposure measures

#### Alcohol outlet access

Data on geocoded locations of all premises licensed to sell alcohol were obtained from the Victorian Commission for Gambling and Liquor Regulation (VCGLR) in September 2014 to coincide with the timing of READI Wave III data collection [23]. The VCGLR approve all licenses for premises supplying alcohol for purchase in Victoria and the website is routinely updated with new licenses. Five categories of alcohol outlets were considered: 1) off-site; 2) on-site; 3) on-site excluding those with late night licenses; 4) on-site with late night licenses; 5) all outlets. On-site excluding those with late night licenses and on-site with late night licenses were subsets of on-site licenses. On-site outlets included outlets with an on-premise or general license from the VCGLR, such as pubs and wine/cocktail bars. Late night licenses permitted consumption beyond 1 am. Off-site premises included outlets granted a packaged liquor license by the VCGLR, allowing the supply of liquor in sealed containers for consumption off-site, such as independent and chain bottle shops, licensed supermarkets, and delicatessens. Given the potential for increased risk associated with some license types, these were considered both combined and independently in analysis.

Outlet addresses were mapped using ArcGIS v10.2 [24]. The full home addresses of READI participants were geocoded to enable access measures to be derived. For each of the alcohol outlet groups, the number of outlets accessible within 0.4 kilometre﻿ (km) and 3 km road network distances of each home address were calculated. Various distances have been used by studies examining access to alcohol outlets and health [13, 15] and the definition of what constitutes an accessible distance is unclear. These two distances were selected since having a large number of outlets within a short distance of home may create an anti-social environment and thus have an effect on the mental health of residents. A larger exposure distance of 3 km may be more appropriate when considering distances travelled to access packaged alcohol. As a 1 km distance threshold has been used elsewhere [25, 26], this was considered in sensitivity analyses.

### Potential confounders

Based on previous literature, participant age, marital status (never married/married or committed relationship/separated, divorced or widowed), children in the household (no/yes), education (did not complete high school/completed high school, trade certificate, or diploma/completed tertiary education), employment status (not employed/employed part-time/employed full-time) and weekly household income (<$500/$500–< $700/$700–< $1000/$1000–< $1500/≥$1500) were considered as potential individual-level confounders of associations between alcohol outlet access and alcohol intake, as well as between alcohol outlet access and depressive symptoms score and depression risk. Urban/rural location was included as a potential area-level confounder. As discussed by Fleischer and Diez Roux [27], variables such as household income and education are not assumed to cause access to neighbourhood exposures, such as alcohol outlets, but are considered to be potential determinants of the probability of living in areas with certain built environment attributes, such as higher levels of alcohol access.

### Statistical analysis

Separate multinomial models were fitted to examine associations between each alcohol outlet access variable and each alcohol intake measure. Robust clustered standard errors were used to take into account the clustered sampling design as participants were sampled from within suburbs. Fractional polynomials were considered [28], some with a zero spike, for the alcohol outlet exposure variables to deal with potential non-linearities in associations with alcohol intake. A zero spike was considered as many participants had no outlets accessible within 0.4 km. The coefficient for the zero spike represents the difference in the expected value of the outcome between those who were unexposed at this distance threshold and those with at least one outlet present, while the coefficient for the continuous term relates only to increasing values for those who had at least one outlet present. By using the zero spike, we were able to simultaneously model both the continuous distribution and the excess zeros.

Linear regression models were fitted to examine associations between alcohol outlet access and continuous depressive symptoms score, using robust standard errors as above. Again, fractional polynomials were used. As depressive symptoms score followed a skewed distribution, a square-root transformation was adopted for this outcome. Logistic regression models were fitted to examine associations between alcohol outlet access and the dichotomised classification ‘at risk’ of depression. All models adjusted for education, employment status household income and urban/rural classification; confounders identified in the literature.

Under the assumption that the missing covariate data were Missing At Random (MAR), we conducted multiple imputation using chained equations (20 imputations) to deal with missing confounder data. The proportion of missing data for each variable is presented in Table 1. In total, 781 (78.5%) participants had complete data for the variables considered. Our imputation model included the outcome variables and all potential confounders described previously, in addition to auxiliary variables (household income, education and employment at Wave I and II; personal income Wave I, II and III).

**Table 1:** Descriptive characteristics of the READI participants at Wave III (*N* = 995)

Alcohol consumption	
Consume within short-term harm guidelines, *n* (%)	
Don’t drink	248 (24.9%)
Yes (≤4 glasses/drinking occasion)	617 (62.0%)
No (>4 glasses/drinking occasion)	130 (13.1%)
Consume within long-term harm guidelines, *n* (%)	
Don’t drink	248 (24.9%)
Yes (≤2 glasses/drinking occasion)	456 (45.8%)
No (>2 glasses/drinking occasion)	291 (29.2%)
Frequency of long-term harm consumption^a^, *n* (%)	
Don’t drink	248 (24.9%)
Infrequent drinker within guidelines (≤2 glasses/drinking occasion less than once/week)	240 (24.1%)
Infrequent drinker above guidelines (>2 glasses/drinking occasion less than once/week)	79 (7.9%)
Frequent drinker within guidelines ≤2 glasses/drinking occasion more than once/week)	216 (21.7%)
Frequent drinker above guidelines >2 glasses/drinking occasion more than once/week)	121 (12.2%)
Missing	91 (9.2%)
Depressive symptoms	
CESD-10 score	
Median (IQR^b^)	7 (3–11)
Minimum-Maximum	0–30
At risk of depression, *n* (%)	
No	667 (67.0%)
Yes	328 (33.0%)
Alcohol outlet access	
Number of outlets within 0.4 km	
Median (IQR)	0 (0–0)
Minimum-Maximum	0–38
Number of outlets within 3 km	
Median (IQR)	10 (5–17)
Minimum-Maximum	0–822
Number of off-site outlets within 0.4 km	
Median (IQR)	0 (0–0)
Minimum-Maximum	0–4
Number of off-site outlets within 3 km	
Median (IQR)	5 (1–9)
Minimum-Maximum	0–90
Number of on-site outlets within 0.4 km	
Median (IQR)	0 (0–0)
Minimum-Maximum	0–45
Number of on-site outlets within 3 km	
Median (IQR)	5 (2.5–9)
Minimum-Maximum	0–740
Number of on-site (excl. late night) outlets within 0.4 km	
Median (IQR)	0 (0–0)
Minimum-Maximum	0–19
Number of on-site (excl. late night) outlets within 3 km	
Median (IQR)	4 (2–8.5)
Minimum-Maximum	0–474
Number of late night on-site outlets within 0.4km^c^	
Median (IQR)	0 (0–0)
Minimum-Maximum	0–18
Number of late night on-site outlets within 3 km	
Median (IQR)	1 (0–2)
Minimum-Maximum	0–266
Other characteristics	
Age (years)	
Mean (SD)	42.2 (7.1)
Minimum-Maximum	23.3–54.4
Relationship status, *n* (%)	
Married/de-facto	741 (74.5%)
Never married	157 (15.8%)
Separated/divorced/widowed	90 (9.0%)
Missing	7 (0.7%)
Children in household, *n* (%)	
No	338 (34.0%)
Yes	656 (65.9%)
Missing	1 (0.1%)
Education, *n* (%)	
Less than high school	209 (21.0%)
High school/trade certificate/diploma	450 (45.2%)
Tertiary	332 (33.4%)
Missing	4 (0.4%)
Employment, *n* (%)	
Full-time	388 (39.0%)
Part-time	376 (37.8%)
Not in employment	217 (21.8%)
Missing	14 (1.4%)
Household income (per week), *n* (%)	
< $500	55 (5.5%)
$500 to < $700	71 (7.1%)
$700 to < $1000	141 (14.2%)
$1000 to < $1500	199 (20.0%)
≥ $1500	322 (32.4%)
Missing	207 (20.8%)
Location, *n* (%)	
Urban	451 (45.3%)
Rural	544 (54.7%)

*Note*: Nine participants had more than 10 outlets (between 17 and 38) accessible within 0.4 km of home. Seventeen participants (including these nine) had more than 200 outlets (between 203 and 822) within 3 km of home. These participants were all located within suburbs located within close proximity to Melbourne Central Business District

^a^Only 904 participants provided both frequency and typical amount consumed

^b^IQR = inter-quartile range

^c^Within rural suburbs, only 3 individuals had a late-night licensed outlet within 0.4 km of home

All analyses were conducted using Stata version 12.0.

## Results

READI participants were aged between 23 and 54 years at Wave III (Table 1). While 33.4% (*n* = 332) women had tertiary education at Wave III, 21% (*n* = 209) reported having less than high school level education. At this wave, 29% (*n* = 291) of women surveyed reported consuming quantities above the risk of long-term harm on a usual drinking occasion in the past 12 months, with 13% (*n* = 130) reporting consumption of quantities above the risk of short-term harm. Approximately 12% of participants reported that they frequently consumed quantities of alcohol above the long-term harm guidelines.

Almost 85% of participants had no alcohol outlets within a 0.4 km network distance of home, while only 5% of participants had no outlets within 3 km.

### i) Alcohol outlet access and alcohol intake

Results from multinomial regression models examining associations between alcohol outlet access and alcohol intake (short-term harm and long-term harm guidelines) are presented in Table 2. Using fractional polynomials, a linear association between alcohol outlet access and alcohol intake was found to provide the best fit to the data. In general, there was no evidence of an association between access to alcohol outlets within 0.4 km and either alcohol intake outcome.

**Table 2:** Associations between alcohol outlet access and usual alcohol intake on a single occasion from multinomial regression (*N* = 995)

	SHORT-TERM	HARM	LONG-TERM	HARM
	Don’t drink vs. Above guidelines	Within guidelines vs. Above guidelines	Don’t drink vs. Above guidelines	Within guidelines vs. Above guidelines
Alcohol outlet exposure^a^	OR^b^ (95% CI)	OR (95% CI)	OR (95% CI)	OR (95% CI)
All outlets				
Number within 0.4 km				
*Zero spike*	0.93 (0.53, 1.62)	0.87 (0.56, 1.35)	0.93 (0.47, 1.82)	0.90 (0.52, 1.55)
*Greater than zero* ^c^	0.98 (0.95, 1.01)	1.00 (0.97, 1.04)	0.96 (0.92, 1.00)*	0.98 (0.94, 1.02)
Number within 3 km	0.996 (0.990, 1.002)	1.000 (0.999, 1.000)	0.995 (0.989, 1.001)	0.999 (0.998, 1.000)**
Off-site outlets				
Number within 0.4 km				
*Zero spike*	0.73 (0.40, 1.33)	0.67 (0.40, 1.13)	0.87 (0.42, 1.80)	0.90 (0.47, 1.75)
*Greater than zero*	0.80 (0.49, 1.30)	0.70 (0.40, 1.24)	0.74 (0.41, 1.34)	0.69 (0.38, 1.26)
Number within 3 km	0.974 (0.953, 0.997)*	1.004 (0.991, 1.016)	0.962 (0.939, 0.986)**	0.988 (0.976, 1.001)
On-site outlets				
Number within 0.4 km				
*Zero spike*	1.22 (0.72, 2.07)	1.08 (0.67, 1.74)	1.32 (0.74, 2.38)	1.17 (0.72, 1.91)
*Greater than zero*	0.97 (0.93, 1.01)	1.00 (0.98, 1.03)	0.96 (0.91, 1.01)	0.99 (0.95, 1.02)
Number within 3 km	0.996 (0.989, 1.002)	0.999 (0.998, 1.000)	0.995 (0.988, 1.001)	0.998 (0.997, 0.999)**
On-site (excl. late night) outlets				
Number within 0.4 km				
*Zero spike*	1.57 (0.70, 3.53)	1.08 (0.62, 1.88)	1.45 (0.54, 3.94)	0.96 (0.48, 1.91)
*Greater than zero*	1.00 (0.92, 1.08)	1.03 (0.97, 1.09)	0.95 (0.86, 1.04)	0.96 (0.88, 1.05)
Number within 3 km	0.993 (0.982, 1.004)	0.999 (0.997, 1.001)	0.991 (0.980, 1.002)	0.997 (0.996, 0.999)**
Late night on-site outlets^d^				
Number within 3 km				
*Zero spike*	0.993 (0.686, 1.436)	0.914 (0.658, 1.268)	1.182 (0.761, 1.836)	1.157 (0.768, 1.742)
*Greater than zero*	0.991 (0.981, 1.001)	0.998 (0.996, 1.001)	0.988 (0.978, 0.998)*	0.996 (0.992, 0.999)**

*p < 0.05; **p < 0.01; ***p < 0.001. Models adjusted for education, employment, household income and urban/rural classification

^a^Fractional polynomials identified linear relationship between continuous alcohol outlet access and alcohol intake as best fit to the data. Separate models were fitted for each of the alcohol outlet access measures (i.e., number within 0.4 km and number within 3 km)

^b^Odds ratio and 95% confidence interval

^c^Greater than zero is the continuous predictor for all observations greater than zero

^d^Too few individuals had a late night on-site outlet within 0.4 km of home (*n* = 13). Only the number within 3 km was considered as an exposure

Considering the 3 km network distance, few outlet access variables were found to be associated with long-term harm consumption (Table 2). However, we did find some support for an association between the number of off-site alcohol outlets and this outcome. The odds of reporting ‘don’t drink’ compared to consumption above long-term harm guidelines decreased with increasing numbers of off-site outlets, although the reduction in odds were very small (e.g., ORs of 0.996 to 0.999).

We found evidence of associations between the number of alcohol outlets accessible within 3 km from home and short-term harm consumption. Although effects were small, the odds of reporting typical consumption within short-term harm guidelines compared to consumption above these guidelines decreased with increasing numbers of all outlets, as well as increasing numbers of on-site, on-site excluding late night and late night on-site outlets. The odds of reporting ‘don’t drink’ compared to above short-term harm guidelines decreased with increasing numbers of off-site and late night on-site alcohol outlets within 3 km.

Considering the frequency of long-term harm consumption (Table 3), there was some evidence of an association between alcohol outlet access within 0.4 km and alcohol consumption. In particular, results showed lower odds of reporting ‘don’t drink’, infrequent drinker within long-term harm guidelines and frequent drinker within long-term harm guidelines compared to frequent drinker above guidelines with increasing numbers of all outlets within 0.4 km. Results consistently showed evidence of reduced odds of reporting ‘don’t drink’ compared to reporting frequent consumption above guidelines with increasing numbers of alcohol outlets of any of the types considered within 3 km.

**Table 3:** Associations between alcohol outlet access and frequency of alcohol intake above recommended guidelines from multinomial regression (*N* = 904)

	Don’t drink vs. Frequent drinker above guidelines	Infrequent drinker within guidelines vs. Frequent drinker above guidelines	Frequent drinker within guidelines vs. Frequent drinker above guidelines	Infrequent drinker above guidelines vs. Frequent drinker above guidelines
Alcohol outlet exposure^a^	OR^b^ (95% CI)	OR (95% CI)	OR (95% CI)	OR (95% CI)
All outlets				
Number within 0.4 km				
*Zero spike*	0.795 (0.386, 1.637)	0.728 (0.376, 1.410)	0.730 (0.362, 1.472)	0.616 (0.278, 1.362)
* Greater than zero* ^c^	0.939 (0.914, 0.966)***	0.960 (0.923, 0.999)*	0.965 (0.940, 0.990)**	0.928 (0.847, 1.017)
Number within 3 km	0.995 (0.991, 0.999)*	0.998 (0.997, 0.999)***	0.999 (0.998, 1.000)*	0.998 (0.995, 1.001)
Off-site outlets				
Number within 0.4 km				
*Zero spike*	0.672 (0.278, 1.620)	0.574 (0.239, 1.380)	0.612 (0.241, 1.558)	0.661 (0.197, 2.214)
*Greater than zero*	0.841 (0.455, 1.553)	0.131 (0.034, 0.506)**	1.065 (0.615, 1.844)	1.121 (0.486, 2.586)
Number within 3 km	0.964 (0.945, 0.983)***	0.989 (0.971, 1.006)	0.996 (0.979, 1.014)	0.981 (0.948, 1.015)
On-site outlets				
Number within 0.4 km				
*Zero spike*	1.191 (0.632, 2.246)	1.056 (0.547, 2.038)	1.031 (0.537, 1.981)	0.795 (0.391, 1.617)
*Greater than zero*	0.941 (0.903, 0.980)**	0.972 (0.947, 0.999)*	0.973 (0.947, 1.000)*	0.941 (0.884, 1.001)
Number within 3 km	0.995 (0.991, 0.999)*	0.998 (0.997, 0.999)***	0.999 (0.998, 1.000)**	0.998 (0.995, 1.001)
On-site (excl. late night) outlets				
Number within 0.4 km				
*Zero spike*	1.331 (0.457, 3.878)	0.871 (0.392, 1.935)	0.964 (0.368, 2.526)	0.610 (0.233, 1.594)
*Greater than zero*	0.911 (0.834, 0.994)*	0.928 (0.854, 1.008)	0.949 (0.886, 1.017)	0.832 (0.691, 1.003)
Number within 3 km	0.992 (0.985, 0.999)*	0.997 (0.995, 0.998)***	0.998 (0.996, 1.000)*	0.997 (0.993, 1.002)
Late night on-site outlets^d^				
Number within 3 km				
*Zero spike*	0.882 (0.528, 1.475)	0.719 (0.415, 1.247)	0.927 (0.588, 1.461)	0.488 (0.289, 0.827)**
*Greater than zero*	0.987 (0.980, 0.994)***	0.993 (0.990, 0.996)***	0.995 (0.993, 0.998)***	0.991 (0.982, 1.000)

*p < 0.05; **p < 0.01; ***p < 0.001. Models adjusted for education, employment, household income and urban/rural classification

^a^Separate models were fitted for each of the alcohol outlet access measures (i.e., number within 0.4 km and number within 3 km)

^b^Odds ratio and 95% confidence interval

^c^Greater than zero is the continuous predictor for all observations greater than zero

^d^Too few individuals had a late night on-site outlet within 0.4 km of home (*n* = 13). Only the number within 3 km was considered as an exposure

### ii) Alcohol outlet access and depressive symptoms

As with alcohol intake, a linear association was found to provide the best fit using fractional polynomials. There was no evidence of an association between alcohol outlet access within either 0.4 km or 3 km network distance from home and CESD-10 scores (Table 4). However, there was some evidence of associations between each alcohol outlet access measure within 3 km and the odds of being classified as at risk of depression (i.e., CESD-10 scores ≥ 10). Increasing numbers of outlets within 3 km were consistently found to be associated with reduced odds of being at risk of depression. However, effect sizes were generally small, e.g., estimated odds ratio of 0.998 (95% CI: 0.997, 1.000; *p* = 0.01) for the effect of the number of all outlets within 3 km, suggesting the odds of being at risk of depression decreased by 0.2% with every additional license within 3 km.

**Table 4:** Associations between alcohol outlet access and depressive symptoms from linear and log-binomial regression models (*N* = 995)

	√CESD-10 Score^a^	At risk of depression^b^ (No/Yes)
Alcohol outlet exposure^c^	β (95% CI)	OR^d^ (95% CI)
All outlets		
Number within 0.4 km		
*Zero spike*	0.098 (−0.108, 0.303)	1.341 (0.851, 2.114)
*Greater than zero* ^e^	−0.003 (−0.013, 0.007)	0.972 (0.933, 1.013)
Number within 3 km	−0.0001 (−0.0004, 0.0002)	0.998 (0.997, 1.000)*
Off-site outlets		
Number within 0.4 km		
*Zero spike*	0.165 (−0.077, 0.407)	1.20 (0.70, 2.05)
*Greater than zero*	0.119 (−0.059, 0.297)	0.73 (0.47, 1.15)
Number within 3 km	−0.0006 (−0.0041, 0.0030)	0.982 (0.967, 0.997)*
On-site outlets		
Number within 0.4 km		
*Zero spike*	−0.002 (−0.009, 0.005)	1.29 (0.87, 1.92)
*Greater than zero*	−0.001 (−0.174, 0.172)	0.98 (0.96, 1.01)
Number within 3 km	−0.0002 (−0.0007, 0.0003)	0.998 (0.997, 0.999)**
On-site (excl. late night) outlets		
Number within 0.4 km		
*Zero spike*	0.105 (−0.143, 0.353)	1.64 (0.95, 2.86)
*Greater than zero*	0.003 (−0.017, 0.023)	0.99 (0.94, 1.05)
Number within 3 km	−0.0003 (−0.0008, 0.0003)	0.997 (0.995, 0.999)**
Late night on-site outlets^f^		
Number within 3 km		
*Zero spike*	0.0450 (−0.1105, 0.2005)	1.360 (1.000, 1.851)
*Greater than zero*	−0.0002 (−0.0009, 0.0005)	0.996 (0.994, 0.998)**

*p < 0.05, **p < 0.01, ***p < 0.001. Models adjusted for education, employment, household income and urban/rural classification

^a^Square root transformation of CESD-10 to deal with skewed distribution

^b^CESD-10 score of 10 or more classed as ‘at risk’

^c^Separate models were fitted for each of the alcohol outlet access measures (i.e., number within 0.4 km and number within 3 km)

^d^Odds Ratio (OR) and 95% Confidence Interval (CI)

^e^Greater than zero is the continuous predictor for all observations greater than zero

^f^Few participants had a late night on-site outlet within 0.4 km so only the 3 km distance was considered for this exposure

### iii) Sensitivity analyses

There was some evidence of associations between alcohol outlet access measures at the 1 km distance threshold and alcohol intake consistent with those identified at 3 km (data not shown). The odds of consuming above short-term harm guidelines compared to within short-term harm guidelines increased with increasing numbers of all, on-site, on-site excluding late night and late night on-site outlets. Consistent with the 3 km distance analysis, we found evidence of reduced odds of reporting ‘don’t drink’ compared to frequently drink above long-term harm guidelines with increasing numbers of outlets for most alcohol outlet types. However, this association was not observed for off-site outlets at this threshold. In general, we found no evidence of an association between access to alcohol outlets within 1 km and either depressive symptoms outcome. Although, increasing numbers of packaged alcohol outlets within 1 km was found to be associated with reduced odds of being at risk of depression, consistent with the 3 km threshold analysis.

Results were similar when considering a complete case analysis (i.e., excluding participants missing data on any of the covariates) and when including those who had moved house during the READI study but remained in a READI suburb to Wave III (data not shown).

## Discussion

Numerous studies have considered associations between access to alcohol outlets and alcohol intake, with some providing evidence that increased access is linked to increased intake [13], although the consistency of evidence has been contested [15]. In our study of women residing in socioeconomically disadvantaged areas of Victoria, we generally found no evidence of an association between alcohol outlet access and alcohol intake when considering a typical consumption threshold of more than two alcoholic drinks. However, our results found associations, albeit small, when considering the four drink threshold, in addition to the frequency of consuming more than two alcoholic drinks. These results showed the odds of consumption at short-term harm levels increased with increasing numbers of outlets within 3 km. Further, the odds of frequent consumption at long-term harm levels increased with increasing numbers of outlets within both 0.4 km and 3 km. However, typically effect sizes were small. These associations are in agreement with other Australian studies which found evidence of an association between access and harmful alcohol consumption [18, 25] but contrast with findings from a study of women in the US which found no evidence of an association between alcohol outlet density (either off-site or on-site) and levels of alcohol intake [29], although their sample was not directly comparable to ours (e.g., it included only young females).

We found no evidence of an association between any of the outlet access measures and CESD-10 score. However, increasing numbers of outlets within 3 km were found to be associated with slightly reduced odds of being classified as at risk of depression. Our results contrast those of a study conducted in Western Australia which examined associations between alcohol outlet access and mental health outcomes, including depression [18], which found evidence of increased odds of hospital contact for a mental health disorder with increased access to alcohol outlets. Although both studies considered associations between alcohol outlet access and mental health outcomes in an Australian context, they are not directly comparable for a number of reasons. Our study was restricted to considering depressive symptoms according to the CESD-10 classification while Pereira et al. considered both self-report of prior medical diagnosis with anxiety, stress and depression analysed as a single outcome showing negligible evidence of an association, in addition to hospital admissions for these conditions using International Classification of Diseases codes; our study only considered women residing in socioeconomically disadvantaged neighbourhoods in Victoria while Pereira et al. included both men and women from various neighbourhoods across Perth; our study considered different categories of alcohol outlet type while Pereira et al. considered only off-premise alcohol outlets, although results from our analysis of off-site alcohol outlets was not in agreement with their findings. As we speculated that increased alcohol outlet could be linked to poorer mental health outcomes, our findings that increased access was associated with reduced odds of being at risk of depression appear counter-intuitive. However, it may be that increased access to alcohol outlets could be protective by providing an opportunity for individuals in the neighbourhood to connect socially. Alternatively, residents in areas with higher numbers of alcohol outlets available may also have access to other community resources which provide opportunities for interaction. Of course, it is important to bear in mind that these could be chance findings as we did not adjust for multiple testing so the results should be interpreted cautiously.

### Limitations

Our study has various limitations. Participants who took part in Wave III of the study were healthier than those who participated at baseline, with a higher percentage (21% compared to 13%) reporting drinking >4 drinks on a typical drinking occasion at baseline and a higher percentage classified as being at risk of depression (36% compared to 33%). Thus greater alcohol intake, and being at risk of depression to a lesser extent, are under-represented. Furthermore, a substantial number of participants dropped out of the study between Wave I and Wave III so these findings may not be generalisable to the wider population of women living in socioeconomically disadvantaged areas in Victoria. As participants had to reside in a READI suburb at each wave of the study, it may be that our sample was depleted due to participants relocating outside of these suburbs. Therefore, we restricted our analysis to those who had remained at the same home address throughout the study period. A further limitation is that all measures are based on self-report questionnaires so responses may be subject to socially desirable reporting. This study, like most others in this area of research, was based on cross-sectional data so it is not possible to determine the direction of associations. It may be that people who consume greater quantities of alcohol choose to reside in neighbourhoods with greater opportunities to purchase alcohol. This is difficult to address when examining built environment effects on health outcomes without long-ranging data. Other limitations were that we only considered access around each participant’s home address. It may be that factors such as exposure to outlets around the workplace are more indicative of increased alcohol consumption, for example. Furthermore, we were unable to verify the locations of the alcohol outlets as recommended elsewhere [15]. In this analysis, we have assumed that the liquor licence registry provide up to date measures of those outlets present on the ground. We also do not have information about where alcohol consumption took place so it is difficult to isolate the effect of the local neighbourhood environment. Furthermore, we only considered the number of alcohol outlets when considering access to alcohol. It may be that other factors, such as marketing, lower retail pricing, and longer hours, are important to consider when examining alcohol accessibility. Finally, it is important to acknowledge that the causes of depression are increasingly recognised as being multifactorial, with social, environmental, psychological and biological contributors [30]. Therefore, any potential associations with built environment measures, such as alcohol outlet access, are likely to be small in magnitude.

## Conclusions

In conclusion, our study found some evidence to support an association between increasing alcohol outlet densities of all types and harmful levels of alcohol consumption, and the association appears to be dependent on the distance threshold considered, among women residing in socioeconomically disadvantaged neighbourhoods within Victoria, Australia. However, higher numbers of alcohol outlets appear to be associated with a lower risk of depression, with further research needed to identify the direction and mechanisms underlying this association. If confirmed, findings warrant consideration in community initiatives aimed at reducing unhealthy alcohol consumption.

## Abbreviations

CESD-10: 10-item Center for Epidemiologic Studies Depression Scale
CI: Confidence interval
IRSD: Index of relative socioeconomic disadvantage
km: Kilometre
MAR: Missing at random
READI: Resilience in Eating and Activity Despite Inequality
SEP: Socioeconomic position
VCGLR: Victorian Commission for Gambling and Liquor Regulation
